# Assessing the uptake of cervical cancer screening among women aged 25-65 years in Kumbo West Health District, Cameroon

**DOI:** 10.11604/pamj.2019.33.106.16975

**Published:** 2019-06-12

**Authors:** Layu Donatus, Fanka Kifu Nina, Dohbit Julius Sama, Claude Ngwayu Nkfusai, Fala Bede, Joyce Shirinde, Samuel Nambile Cumber

**Affiliations:** 1 Department of Reproductive Health, Faculty of Health Sciences, Catholic University of Central Africa, Yaounde, Cameroon; 2 Department of Microbiology and Parasitology, Faculty of Science, University of Buea, Cameroon; 3 Cameroon Baptist Convention Health Services (CBCHS), Yaounde, Cameroon; 4 School of Health Systems and Public Health, Faculty of Health Sciences, University of Pretoria Private Bag X323, Gezina, Pretoria, South Africa; 5 Faculty of Health Sciences, University of the Free State, Bloemfontein, South Africa; 6 Section for Epidemiology and Social Medicine, Department of Public Health, Institute of Medicine, The Sahlgrenska Academy at University of Gothenburg, Gothenburg, Sweden

**Keywords:** Uptake, cervical cancer, screening, health district

## Abstract

**Introduction:**

Cervical cancer remains one of the leading health hazards affecting a majority women across the globe. The situation is even more, preoccupying particularly in areas where screening programmes and services are absent. The World Health Organization (WHO) says “cervical cancer is the fourth most frequent cancer in women, with an estimated 570,000 new cases diagnosed in 2018 which represents 6.6% of all female cancers. Approximately 90% of deaths from cervical cancer occurred in low- and middle-income countries”. Despite the high mortality rate from cervical cancer globally, the trend could be reduced through a comprehensive approach that includes prevention, early diagnosis, effective screening and treatment programmes. In Cameroon, the prevalence of cervical cancer is 24% among women of reproductive age. An estimated 1,993 new cases are recorded annually in Cameroon with 1676 deaths. Despite this precarious situation, the uptake in cervical cancer screening service remains poor and stands at 19.6% in Cameroon. It is against this background that this paper evaluates the uptake of cervical cancer among women aged 25-65 years in the Kumbo West Health District (KWHD). Specifically, this study assesses the knowledge of women in this health district on cervical cancer and determines factors that affect the uptake of cervical cancer screening services.

**Methods:**

This study is a cross-sectional study in the KWHD involving 253 consented women between the ages 25 to 65 years. The principal research instrument was a three-part questionnaire designed to collect information on socio-demographic profile, cervical cancer knowledge and associated factors for uptake in cervical cancer screening. Data was entered in MS Excel and analysed using Excel. Results were presented in tables and figures.

**Results:**

Our study reveals that a majority of the participants (74.70%) had heard of cervical cancer and 43.48% had undergone cervical cancer screening. Again, 24.51% and 29.25% of the participants respectively could not identify any risk factor and symptom of cervical cancer.

**Conclusion:**

The study revealed that the uptake of cervical cancer screening in KWHD is higher than the national uptake. The level of awareness on the risk factors and symptoms of cervical cancer is low, posing a need to put more emphasis on educating and creating awareness of cervical cancer among communities on risk factors, prevention measures and signs and symptoms in all the health areas of the KWHD.

## Introduction

Cervical cancer is a malignant proliferation of the cells of the uterine cervix and can be treated if diagnosed earlier. Cervical cancer occurs when abnormal cells in the lining of the cervix grow in an uncontrolled way [1]. The cervix is the lower part of the womb. It is the opening to the vagina from the womb. The main symptom of cervical cancer is unusual bleeding from the vagina. Finding changes in the cells through screening can help to prevent cancer from developing. Cancer is among the leading causes of death worldwide and accounted for 8.2 million deaths in 2012. Projections based on the GLOBOCAN 2012 estimates, predict a substantive increase of 19.3 million new cancer cases per year by 2025, due to the growth and ageing of the global population [1]. Cervical cancer (CC) is a disease that occurs when cells of the cervix are infected with the human papillomavirus (HPV). In 2012, 528 000 new cases of cervical cancer were diagnosed worldwide; of these, about 85% occurred in less developed countries. In the same year, 266 000 women died of cervical cancer worldwide; almost 9 out of every 10 of these, or 231,000 women in total, lived and died in low- to middle- income countries. It is the leading cause of cancer deaths among women in Sub-Saharan Africa (SSA) with approximately 34.8 new cases diagnosed annually per 100,000 women and 22.5 deaths per 100,000 annually [2]. In Africa, there are 99,038 new cases with 60,098 deaths annually. It is the second leading cause of death from cancers among women aged between 14-44 years. Cervical cancer is a major problem in South Africa and it is the second most common cancer and affects one out of every 41 women. In 2009, estimates revealed that 43 000 women were diagnosed with cervical cancer and 274 000 died from the disease in South Africa [3]. On her part, in 2014 Ethiopia had about 27.19 million women above the age of 15years projected to be at risk of developing cervical cancer. Current assessments indicate that, an estimated 7,095 cases of cervical cancer and 4,732 deaths resulting from it occur every year [4]. In Kenya, the estimated annual number of cervical cancer cases stands at 2,454 cases, while the annual number of deaths from cervical cancer sums up to 1,676 and accounts for 70-80% of all cancers of the genital tract. Cervical cancer screening coverage in Kenya for all women within the ages 18 to 69 years is only 3.2% [5].

In Cameroon, Cervical cancer (CC) is the second most encountered cancer in women after breast cancer with 7.1million women with ages 15 years and above who are at risk of developing the diseases. Cervical cancer is the 2^nd^ leading cause of cancer deaths in women aged 15 to 44 years in Cameroon [5]. According to the National Committee for the Fight against Cancers, Cervical Cancer accounts for 24% of female cancers [6]. In Cameroon, about 1,993 new cervical cancer cases are documented with 1,120 cervical cancer deaths [7]. In 2010, 30% of deaths among women were attributed to cervical cancer. The cervical cancer registry in Yaoundé estimates incidence at 107 cases for 100,000 inhabitants [8]. The epidemiology rightfully points out the dire situation of the morbidity and mortality from cervical cancer beginning from global, to regional and country with the need for primary prevention interventions (screening and treatment of precancerous lesions). Screening techniques like the Pap smear, VILI/VIA and cytology are available in Cameroon. Despite all these challenges as identified from literature associated with the low uptake, there is low cervical cancer screening uptake in Cameroon. Thus, there is need to question these challenges and the paucity of data collection on cervical cancer. Early diagnosing of cervical cancer and providing access to effective treatment can significantly improve the likelihood of survival. Divine Njodzeven, Coordinator of the Women's Health Programme (WHP) with the CBC Health Services has been doing much in the area of sensitization on cervical cancer in the Northwest Region of Cameroon. He notes that cervical cancer is a public health challenge and a threat to the health of women, with over 2000 cases diagnosed in Cameroon yearly, and with more than half dying. He regrets that though being a preventable and treatable condition, many are not aware of the condition with poor update for cancer screening. “Faced with such, the WHP is very intentional on raising awareness and screening,” avows divine. The CBC Health Services is paying particular attention to secondary level prevention of cervical cancer given that once a woman develops it, it takes millions and lots of resources to treat. The Nkwen Baptist Health Center for example, has a comprehensive approach to preventing cervical cancer which includes community education, vaccination and social mobilization which are approaches in line with WHO recommendation. Noticing that poverty and lack of finances remain one of the barriers to accessing healthcare and services and accounts for the low cancer screening up take, the NBHC during their one-month screening campaign subsidized the cost of screening with a drop from 4000 FCFA during normal consultations to 2000 FCFA during their awareness and screening campaigns.

## Methods

**Study design and research type:** this study is an observational and cross-sectional study.

**Study population and target population:** the target population for this study are women between the ages 25-65 years which is Cameroon's recommended age group for cervical cancer screening.

### Selection criteria

**Inclusion criteria:** women with ages between 25-65 years and who have been residing in the Kumbo Health District for at least two years. Those who met the above criteria and were willing to participate.

**Exclusion criteria:** women who did not give consent to participate; women who wanted to be motivated before they responded to the questionnaire and women that had undergone complete hysterectomy were not included.

**Sample size:** to determine the sample size of this study, the sample size was calculated using the formula:

$$N=\frac{\left(1.96)X0.196(1-0.196\right)}{{\left(0.05\right)}^{2}}$$

N=required sample size, t=level of confidence at 95% (1.96), p= prevalence of studied variable, in this case prevalence of cervical cancer screening in Cameroon 19.6% [6], e: error margin at 5% (value of 0.05) substituting this into the formula: N= (1.96)^2^ x 0.196(1-0.196) / (0.05)^2^. N=242 which we rounded up to 250 participants. We needed at least 250 participants for the study.

**Sampling method:** a stratified random sampling method was used wherein each Health Area represented a stratum. In each Health Area, quarters were selected by simple random sampling, that is, we sampled the second quarter in each Health Area. In each quarter and with the help of the quarter head, houses were selected randomly (every third house) to get the sample size in that quarter. To be able to get a good representative of the community, a member was recruited from each household with priority given to the most elderly person. This was done for all the 11 Health Areas to meet a sample size of 250. The population of the Health Area with respect to that of the district was taken into consideration to choose the number of participants per Health Area. These Health Areas are: BBH, Buh, Kai, Kikaikelaiki, Kikaikom, Kitiwum, Kumbo-CMA, Kumbo-Urban, Kuvlu, Melim and Nkumkov.

**Data collection and analysis:** all participants who consented were interviewed using a structured questionnaire adapted from a questionnaire formulated by Mohammed [9]. Prior to its use in this study, a total of 20 respondents from Kumbo East, an area with similar characteristics as KWHD were solicited with the aim of revising poorly structured questions. This was to be done by estimating the average time required to fill the questionnaire, and eventually validating the use of the questionnaire in the context of this study. A total of 253 questionnaires were administered to participants under study for a period of 2 months to assess their uptake of cervical cancer screening among women in the KWHD. Knowledge on uptake of cervical cancer screening consisted 12 questions and each correct response was scored as 1 and 0 for a wrong response. The knowledge scores for an individual was calculated and summed up to give a total knowledge score on 12. A score between 0-4 was classified as poor, 5-8 as good and 9-12 as excellent as adapted from Abongwa's [10] study. The quantitative data was analysed using Microsoft Excel and the results were presented in terms of numbers and percentages in the form of charts and tables. The main questions to access the knowledge of the uptake of cervical were the following.

**Reason for never screening:** a) it may be painful; (b) am not informed, shy to expose my private part; c) i am healthy; d) i am afraid a CCS would reveal cervical cancer; e) it is expensive; f) i haven't just decided.

**Data processing and analysis:** data collected was checked, coded and entered into Microsoft Epi software version 7 and exported to Excel for analysis. It was exported to excel to generate descriptive diagrams, histograms, pie charts, tables to present the percentages and frequencies.

**Ethical consideration:** ethical approval for the study was obtained from the Institutional Research Ethics Committee for Human Health (CIERSH) at the School of Health Sciences of the Catholic University of Central Africa. Administrative clearance was obtained from the Regional Delegation of Public Health, Bamenda, Directorate of the SHS/CUCA and from the District Chief of Service for KWHD. Participants were investigated in strict confidentiality. Written consent was obtained from all participants and confidentiality of participants was ensured by using anonymous questionnaires.

## Results

**The sociodemographic characteristics of respondents:** the mean age of the respondents was 40.92 years. A majority of the respondents with 33.99% (n=86) were between the ages 25-34 years, followed by those with ages between 35-44 years (32.02%), those with ages 45-54 years with 18.58% and those with ages 55-65 years forming a 15.42% of the respondents. A majority of the respondents, 40.71% (n=103) were married, 29.64% (n=75) single and 12.25% (n=31) living together, 11.07% (n=28) widowed, 3.16% (n=8) divorced, and the rest of the respondents 3.16% (n=8) were separated. Out of all the women interviewed, 19.76% (n=50) had primary level education, 42.29% (n=107) had secondary level education, 26.09% (n=66) had university level education, and 11.86% (n=30) of the respondents had never had any formal education. Most of the participants 44.66% (n=113) earned less than 25,000 FCFA per month, 14.23% (n=36) earned between 25,000-49,999, while 12.25 % (n=31) earned between 50,000-100,000, 7.11% (n=18) had no monthly income, where as 15.42% (n=39) earned more than 100,000 FCFA per month. In determining the parity of the respondents, most of them 47.04% (n=119) had given birth at least 1-4 times, 29.64% (n=75) from five times and above while the rest 23.32% (n=59) have never given birth (Table 1).

**Table 1:** Socio-demographic characteristics of participants

Variable	Category	Frequency	Percentage
Age (Years)	25 – 34	86	33.99
	35 – 44	81	32.02
	45 – 54	47	18.58
	55 – 65	39	15.42
	**Total**	**253**	**100**
Marital status	Single	75	29.64
	Married	103	40.71
	Separated	8	3.16
	Divorced	8	3.16
	Living together	31	12.25
	Widow	28	11.07
	**Total**	**253**	**100**
Education	None	30	11.86
	Primary	50	19.76
	Secondary	107	42.29
	University	66	26.09
	**Total**	**253**	**100**
Monthly Income (FCFA)	No income	18	7.11
	< 25,000	113	44.66
	25,000 – 49,999	36	14.23
	50,000-100,000	31	12.25
	>100,000	39	15.42
	Not Stated	16	6.32
	**Total**	**253**	**100**
Parity	0 Children	59	23.32
	1 - 4 Children	119	47.04
	≥ 5 Children	75	29.64
	**Total**	**253**	**100**

**Knowledge on cervical cancer:** the majority of the respondents 74.70% (n=189) had heard of cervical cancer, whereas 25% (n=64) had never. Evaluating the source of awareness, a majority of the respondents n=80 (31.6%) revealed that they had heard about cervical cancer from the HCW, followed by the mass media n=39 (15.4%), families and friends n=38 (15.1%) with printed materials and teachers as source of information being the least sources of information n=10 (3.9%) on cervical cancer.

**Figure 1:**
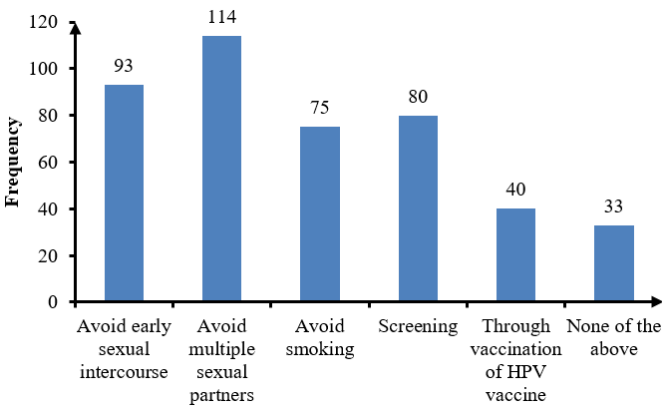
Methods of prevention of cervical cancer

**Knowledge of signs and symptoms, risk factors and prevention modes of cervical cancer:** among the respondents, 43.08% identified having multiple partners as the highest risk factors, while acquiring HPV was scored as the least risk factor with a 22.13% whereas 24.51% of the respondents were unable to identify a single risk factor. In assessing the symptoms of cervical cancer, abdominal pain was identified as being the highest symptom of cervical cancer by the respondents with a 44.66% response, followed by provoked vaginal bleeding with 37.94% response rate, whereas 20.25% could not identify any symptom of cervical cancer. As a means of preventing cervical cancer, the respondents pointed out that avoiding multiple sexual partners was essential 25.6% (n=114), followed by avoiding early sexual intercourse with 36.76%, screening with 31.62%, where as 13.04% could not identify a preventive method (Figure 1, Table 2).

**Table 2:** Distribution of risk factors, symptoms and prevention modes of respondents

VARIABLE	Frequency	Percentage
Heard about cervical cancer	189	74.70
Screening procedures to detect CC	220	86.96
**Risk Factors**		
Having multiple partners	109	43.08
Early intercourse	99	39.13
Cigarette smoking	85	33.60
HIV infection	67	26.48
Acquiring HPV	56	22.13
None of these factors	62	24.51
**Symptoms of CC**		
Abdominal pain	113	44.66
Provoked vaginal bleeding	96	37.94
Vaginal foul smelling discharge	73	28.85
None of these symptoms	74	29.25
**CC prevention**		
Avoid early intercourse	93	36.76
Avoid multiple partners	114	45.06
Avoid smoking	75	29.64
Screening	80	31.62
Vaccination with HPV vaccine	40	15.81
No prevention method	33	13.04

**Awareness of procedures to detect cervical cancer, uptake of cervical cancer among respondents and duration of last screening:** a majority of the respondents 86.96% (n=220) believed there were procedures to detect cervical cancer while 13.04% (n=33) did not believe there were screening procedures to detect cervical cancer. Cervical cancer screening uptake among the respondents was found to be 43.48% (n=110). Majority of the respondents had never been screened for cervical cancer 56.52%. Among those who had been screened, 48.18% had done it more than three years ago (Table 3).

**Table 3:** Duration of last screening

Variable	Attribute	Frequency (%)
When were you lastly screened	Within last three years	57 (50.89)
	More than three years ago	53 (48.18)
	Total	110 (100)

**Determining factors to cervical cancer screening uptake:** a total of 77.47% of the respondents knew a health care unit to seek screening while 22.53% had no idea of where to get screened. A greater percentage of the respondents 59.68% (n=151) had no idea on the cost of screening while 17.39% respondents said it was more than 5000fcfa and 22.92% responded that it was less than 5000fcfa. In assessing the distance to a screening unit, a majority 42% of the participants had to cover a distance more than 5km to assess a screening unit while 24% had no idea on the distance covered and a slightly higher portion of the respondents 35% covered a distance less than 5km (Table 4).

**Table 4:** Availability/affordability of screening services

Variable	Category	Frequency	Percentage
Estimated cost of CCS	More than 5,000Fcfa	44	17.39
	Less than 5,000Fcfa	58	22.92
	No idea	151	59.68
	Total	253	100
Health care unit for CCS	Knows a unit	196	77.47
	No idea	57	22.53
	**TOTAL**	253	100

**Reasons for not screening for cervical cancer:** in evaluating reasons for not haven done cervical cancer screening among those who have never screened, a majority of the respondents 25.30% (n=64) responded that they were not informed. This was followed by 9.49% made up of those who said they haven't decided yet and 6.32% made up of those who claimed the screening is expensive. While, 5.53% opined that they were healthy and saw no need for screening, 4.35% thought it could be painful, 3.16% were shy to expose their private body parts and 2.37% feared the CCS would reveal cervical cancer (Table 5).

**Table 5:** Reasons for not screening for cervical cancer

Reason for never screening	Frequency	Percentage
It may be painful	11	4.35
Am not informed	64	25.30
Shy to expose my private part	8	3.16
I am healthy	14	5.53
I am afraid a CCS would reveal cervical cancer	6	2.37
It is expensive	16	6.32
I haven’t just decided	24	9.49
TOTAL	143	56.52

**Figure 2:**
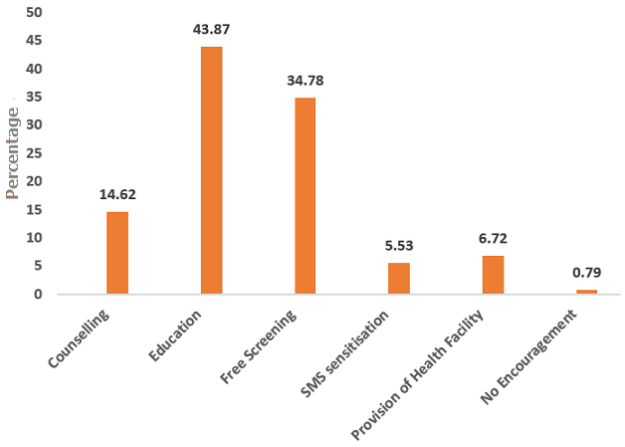
Ways to encourage uptake

**Ways to encourage uptake:** when respondents were asked about what could be done to increase the uptake of cervical cancer screening services, a 43.87% (n=111) majority suggested that education about the disease, followed by free screening 34.78% (n=88), counselling 14.62% (n=37), provision of centers 5.53% (n=14) and SMS sensitization 6.72% (n=17) were ways to encourage uptake (Figure 2).

## Discussion

The mean age of the respondents was 40 years with majority of the respondents 33.99% (n=86) with ages between 25-34 years. There was no relationship between age and uptake of cervical cancer screening. This is higher than the 28.4%; based on a study carried out by Enow and colleagues Yaounde, Cameroon [11]. This is in contrast with other studies that have found lower rates of screening among women aged 20-29 years [12]. Another study done in Kenya on risks and barriers to cervical cancer screening among 219 women attending MNCH-FP clinic at the Moi teaching and referral hospital (MTRH), found that women over 30 years were more likely to have screened for cervical cancer than younger women [12]. A majority of those who had been screened 69.7% (n=54) were married but there was no significant relationship between marital status and uptake of screening. Other studies also revealed that unmarried and widowed women are less likely than the married to obtain screening [12]. These findings are in contrast with other two studies that revealed that single women are more likely than married women to have been screened [13]. There was no relationship observed between level of education and uptake of cervical cancer screening. This contrasts with studies that revealed that women with high screening rates have a higher level of education [12]. A relationship was not observed between employment status and uptake of cervical cancer screening. This contrasts studies carried out in India [14] and at Kenyatta National Hospital (KNH) in Kenya where there was a significant association between employment and CCS uptake. This may explain why 34.78% of those who had not attended screening services recommended free screening as a strategy to increase uptake as these women lack the means to finance their health care needs. A 75% (189) of the women had heard of cervical cancer. This level of awareness is in keeping with a study done in Kenya which found cervical cancer awareness to be at 73.2 % [14] but higher than a study done in India where only 44.5% of respondents were aware of CC [15]. The source of information on cervical cancer screening among most of the respondents were informed by a health care worker (HCW 32% (80)) which ties with a study in Ethiopia where 33% of the respondents got information on cervical cancer from HCW [16].

Women's awareness on cervical cancer screening was significantly associated with uptake of screening or screening status. This means that women who are aware of cervical cancer and screening are more likely to undergo screening for the disease. This ties with of the outcome of a study carried out in North Central Nigeria were 88.9% of women were aware of CCS with a screening rate of 8.0%. The proportion of those who had screened were from those who were aware [3]. This was contrary to a study carried out in South Western Nigeria where 41.9% were aware of cervical cancer but only 3.3% had undergone a screening. This indicates that the more people are aware of CCS, the more the screening uptake will increase. This study was similar to [17] in Buea Cameroon, where up to 42.2% of participants had poor knowledge on cervical cancer. The awareness on cervical cancer risk factors was also found to be inadequate. Only 22.3% (n=56) were aware that infection with HPV is a risk factor for the development of cervical cancer. About 74.7% of the women had heard about cervical cancer, and 43.48% (n=110) of the respondents had been screened. However, this was higher than the national rate of screening which stood at 19.6%. Other studies done among women reveal low uptake of screening. A study done in Maroua in the Far North Region of Cameroon by Tebeu *et al.*, 2008 revealed a low uptake of screening as 4 of 48 (8.3%) of the participants had been screened [18]. A majority of the respondents were aware of a health unit where they could seek screening as 77.47% knew where to screen and only 22.53% did not know where to get screened. These results are greater than those of a study in India carried out to assess women's knowledge on Cervical Cancer. In this study, only 22% of women knew a center where they could seek screening [19].

A greater proportion of the respondents 59.68% had no idea on the cost of screening but 17.39% thought it was greater than 5000fcfa which is rather less than 5000fcfa in this district. The cost of screening can affect uptake as 6.32% of those who have never screened responded that they have not undergone screening because it could be expensive. Also, this research reveal that there was an association between distance and uptake of cervical cancer screening showing that the more the distance, the less likely the possibility to screen. This was similar to those of a study carried out in Kenya by Morris, 2016 were 68% of the respondents could access a health unit. Long distances to screening center reduces the likelihood of women accessing screening [20]. When those who had never been screened were asked for reasons why they have never been screened, they advanced reasons such as not being informed, not haven decided, cost of screening, feeling healthy, it may be painful and being afraid of having a positive test were the most advanced reasons for not undergoing cervical cancer screening. These reasons are different from those in a study in China by [21, 22] where the major barriers identified by the women as those that influence screening utilization were: anxious feeling of being diagnosed, no symptoms, unawareness of the benefits of screening, CC is incurable even if screening is effective and husband disapproval of screening. Being aware of the cost of screening is important as more than half of the participants had no idea on the cost of screening. Sensitization of the population is important as the more people are aware, the more they will take positive actions concerning their health.

**Study limitations:** the researcher did not recruit participants from all the Health areas of the KWHD. Furthermore, studying self-reported knowledge is itself a limitation because one cannot rely totally on the information provided by the participants because of recall bias and social desirability bias. Despite these shortcomings, this study provides relevant information in the context of very limited epidemiological data on cervical cancer in the KWHD of the North West Region of Cameroon, especially among women in the semi-urban milieu.

## Conclusion

The study revealed a low level of cervical cancer screening among women in the KWHD though higher than the national level. The level of awareness of risk factors and symptoms was low so there is need therefore, to put more emphasis on educating and creating awareness among communities about cervical cancer, risk factors, signs and symptoms in all the Health Areas of the Kumbo west health district. There was a relationship between awareness and uptake of cervical cancer indicating that with awareness, more people can uptake screening. The barriers to screening identified by most participants included not being informed of cervical cancer screening services, health facility related challenges such as distance to health facilities and costs of the service, among other information and continuous sensitization are key tools in enhancing the uptake of cervical cancer screening. Attempts should be made to reach out to women who rarely visit health care services, for example, through increasing health campaigns in partnership with other organizations in the area, media and brochures.

### What is known about this topic

- In Cameroon, 1,993 new cases of cervical cancer and 1,676 deaths occur annually;
- According to the national committee for the Fight against Cancers, it has a 24% prevalence rate and 19.6% screening rate;
- Vaccination against HPV and screening constitute the tools to fighting against cervical cancer in Cameroon with a coverage of 19.7% and contributing to high mortality rates as diagnoses and treatments are not done on time.

### What this study adds

- This paper reveals that the level of cervical cancer screening among women in the KWHD stands at 43.48% higher than that at the national level;
- The study reveals that the level of awareness of the risk factors and symptoms of cervical cancer in KWHDis low. Consequently, there is need to lay more emphasis on educating and creating awareness among communities about cervical cancer, risk factors, signs and symptoms in all the Health Areas of the Kumbo West Health District;
- The study also reveal barriers to cervical cancer screening such as lack of information on cervical cancer screening services, inaccessibility of health facilities, costs of the screening service, individual perceptions such as having no signs and symptoms of the disease, fear of the painful procedure, dread to expose private parts and fear of being detected of having cervical cancer after the test.

## Competing interests

The authors declare no competing interests.
